# Evaluation of singlet oxygen generators of novel water-soluble perylene diimide photosensitizers†

**DOI:** 10.1039/d3ra02338a

**Published:** 2023-05-22

**Authors:** Furkan Özçil, Funda Yükrük

**Affiliations:** a Department of Chemistry, Faculty of Arts & Science, Balikesir University Balikesir 10145 Turkey fyukruk@balikesir.edu.tr

## Abstract

In this study, novel photosensitizers using three water-soluble green perylene diimide (PDI)-based ligands were synthesized, which can be used as photosensitizing drugs in photodynamic cancer therapy (PDT). These three efficient singlet oxygen generators were prepared *via* reactions of three newly designed molecules, namely 1,7-di-3-morpholine propylamine-*N*,*N*′-(l-valine-*t*-butylester)-3,4:9,10-perylyne diimide, 1,7-dimorpholine-*N*,*N*′-(*O-t*-butyl-l-serine-*t*-butylester)-3,4:9,10-perylene diimide and 1,7-dimorpholine-*N,N*′-(l-alanine *t*-butylester)-3,4:9,10-perylene diimide. Although there have been numerous photosensitizers, most of them have a limited useable range of solvent conditions or low photostability. These sensitizers have demonstrated strong absorption and red-light excitation. The singlet oxygen production of the newly synthesized compounds was investigated using a chemical method with 1,3-diphenyl-*iso*-benzofuran as a trap molecule. In addition, they do not have any dark toxicity at the active concentrations. Owing to these remarkable properties, we demonstrate the singlet oxygen generation of these novel water-soluble green perylene diimide (PDI) photosensitizers with substituent groups at the 1 and 7 positions of the PDI material, which are promising for PDT.

## Abstract 2

In this study, novel photosensitizers using three water-soluble green perylene diimide (PDI)-based ligands were synthesized, which can be used as photosensitizing drugs in photodynamic cancer therapy (PDT).

## Graphical abstract

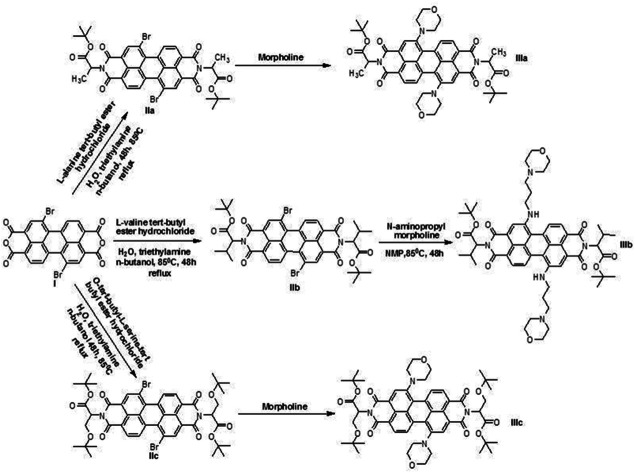

## Introduction

Perylene diimides (PDIs) are reddish dyes, and their derivatives are among the most extensively investigated dyes owing to their high absorption coefficient for visible light, high thermal and chemical stability and high quantum yield. As industrial pigments, PDIs are insoluble in common solvents due to aggregation, and hence their processing, material applications and synthesis remain restricted. Functional PDIs are used extensively in various applications, such as solar cells, dye lasers and photovoltaic devices, with increasing solubility.^1–3^ Also, water solubility is a tricky problem with PDI dyes; however, recently it has been addressed successfully for the PDI molecules as photosensitizers.^4^ There is a major advantage for PDI materials in bio-applications when they are water-soluble and nonaggregating in aqueous media. Various modifications of the parent chromophore have yielded improved solubility characteristics. Most important among these is the substitution at the bay region, the positions of 1, 6, 7 and 12 of the perylene core. Furthermore, appropriate modifications using additional ionic side substitution on the perylene core can increase the water solubility of PDIs and shift the maxima of the absorption spectra of the chromophores up to approximately 750 nm. The ionic substituents can suppress π–π aggregation and shield the inner perylene molecules, thus contributing to the water solubility that is essential for biological applications. Therefore, the water solubility can still be improved further. PDIs have been studied as photosensitizers owing to their high quantum yields of ^1^O_2_ generation.^4^ Photosensitizers with the ability to produce ^1^O_2_ under light irradiation have attracted considerable attention owing to their fascinating applications as represented by PDT. Porphyrins, tetrapyrroles, phytalocyanines and heteroanthracenes are among such singlet oxygen producing photosensitizers.^5–8^ Injectable and photosensitizing supramolecular hydrogels show great potential in biomedical applications, such as PDT and wound dressings.^22^

PDI derivatives have electron acceptor properties and photochemical stability.^9^ Applications of PDIs as induced organic compounds include photochemistry,^10^ photophysics,^11^ molecular switches and wires,^12^ electrochemical donor acceptors,^13^ light-harvesting arrays,^14^ thin films,^15^ organic light-emitting diodes,^16^ photodynamic therapy,^17,18^ living cell staining,^19^ fingermark detection,^20^ biomedical applications,^21,22^ organic nanodevices,^23^ dye lasers,^24^ solar cells,^25^ chemosensors,^26^ photosensitizers,^4^ reprographic processes^27,28^ and photovoltaic devices.^29^

Photodynamic therapy (PDT) is a non-invasive treatment for both cancerous and noncancerous diseases involving the combined use of near-visible or visible light and photosensitizers.^30–32^ The photosensitizer activates ground state oxygen and generates singlet oxygen when excited with light.^33^ PDT is applied with the drug Photofrin (porfimer sodium) in the treatment of various cancers, such as lung, esophageal, bladder, head, neck, skin, perianal, metastatic breast, gynecologic and others.^34^

PDT not only improves the quality of life with adjuvant therapy in advanced cancer cases, but also curatively treats early-stage cancer.

**Fig. 1:**
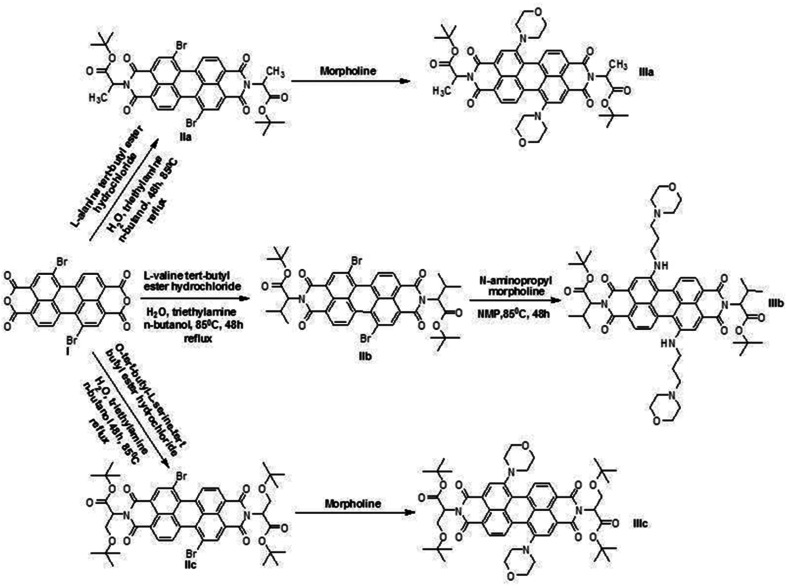
Synthesis of green perylene diimide derivatives. IIIa-1,7-dimorpholine-*N*,*N*′-(l-alanine *t*-butylester)-3,4:9,10-perylene diimide (1), IIIb-1,7-di-3-morpholine propylamine-*N*,*N*′-(l-valine-*t*-butylester)-3,4:9,10-perylene diimide (2), IIIc-1,7-dimorpholine-*N*,*N*′-(*O-t*-butyl-l-serine-*t*-butylester)-3,4:9,10-perylene diimide (3).

**Fig. 2:**
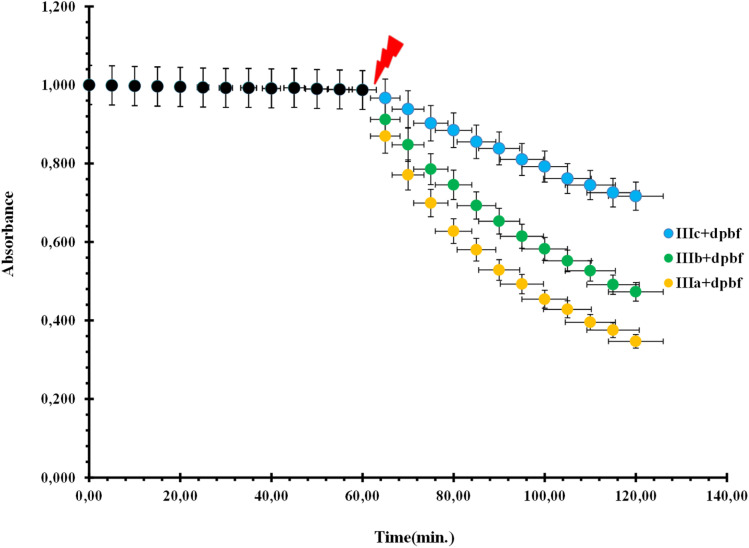
Change in the relative absorbance of 5 × 10^−6^ mM green perylene diimides under red light illumination at 421.5 nm in the presence of 5 × 10^−5^ mM 1,3-diphenyl-*iso*-benzofuran (dpbf); illumination starts after 60 min dark treatment.

As an improvement of the current photodynamic therapy treatment,^4^ we developed novel photosensitizers using green PDIs, which can be excited at longer wavelengths with more penetrating light, because such compounds are highly efficient singlet oxygen generators (Fig. 1). The synthesized novel green PDI photosensitizers have zero cytotoxicity in the absence of light (Fig. 2). This means that tumor damage can be controlled by the amount of light irradiated for a given drug dose in the photodynamic cancer treatment method.

## Results

Three novel water-soluble perylene diimides were studied in isopropanol at 5 × 10^−6^ mM concentration for singlet oxygen generation properties. The experiments were carried out in which the sensitizers were precisely determined. The excitation of PDI dyes was provided by light from 240 W tungsten lamps equipped with a filter to remove light with <600 nm. The light intensity was 11 mW cm^−2^.

**Fig. 3:**
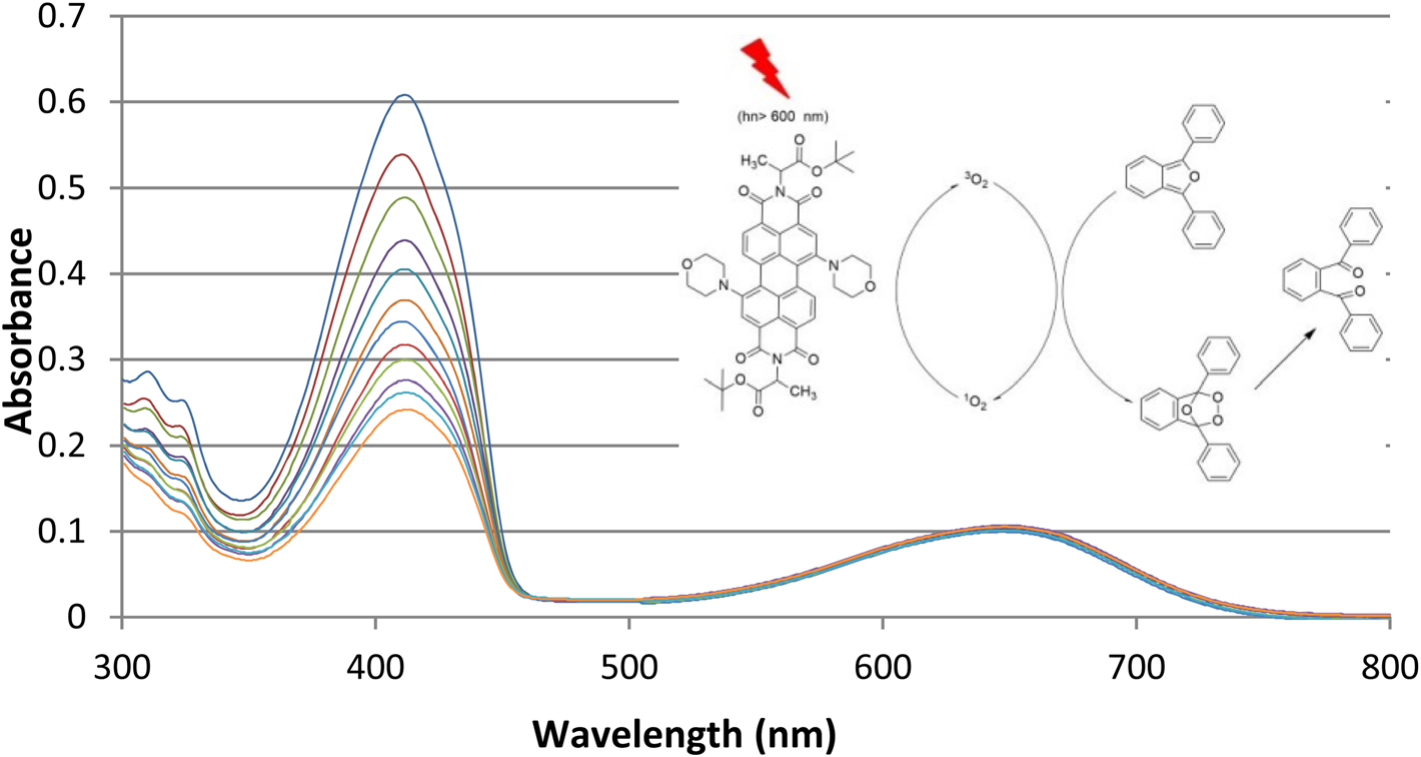
Change in the absorbance spectrum of singlet oxygen trap ‘dpbf’ and compound 1, IIIa-1,7-dimorpholine-*N*,*N*′-(l-alanine *t*-butylester)-3,4:9,10-perylene diimide, on irradiation with light (*λ* > 600 nm).

Before irradiation, the change in the absorption spectrum of the selective singlet oxygen trapping agent 1,3-diphenyl-*iso*-benzofuran (5 × 10^−5^ mM) in the aerated media was measured for 5 min (Fig. 2). Under irradiation, the absorbance was measured every 5 min in the dark and also under red light. PDI photosensitizer solutions showed significant decrease in absorbance, whereas the absorbance at the peak absorbance wavelength of 1,3-diphenyl-*iso*-benzofuran as a trapping molecule (Fig. 3) (400 nm) did not change. We also obtained absorbance data under the same conditions until 60 min in the dark, followed by a 60 min period of irradiation with red light, in order to eliminate any contribution to the absorbance changes from dark reactions (Fig. 2). Under dark conditions, the compounds did not show any difference in the absorbance measurement of the reaction mixture. However, on turning on the light, degradation was initiated by the singlet oxygen trap. The absorption spectrum of one of the compounds in pH 7.4 Tris buffer solution showed broad and red-shifted peaks centered at 655 nm (Fig. 3). In the same buffer solution, the extinction coefficients were near 20 000 M^−1^ cm^−1^.

**Fig. 4:**
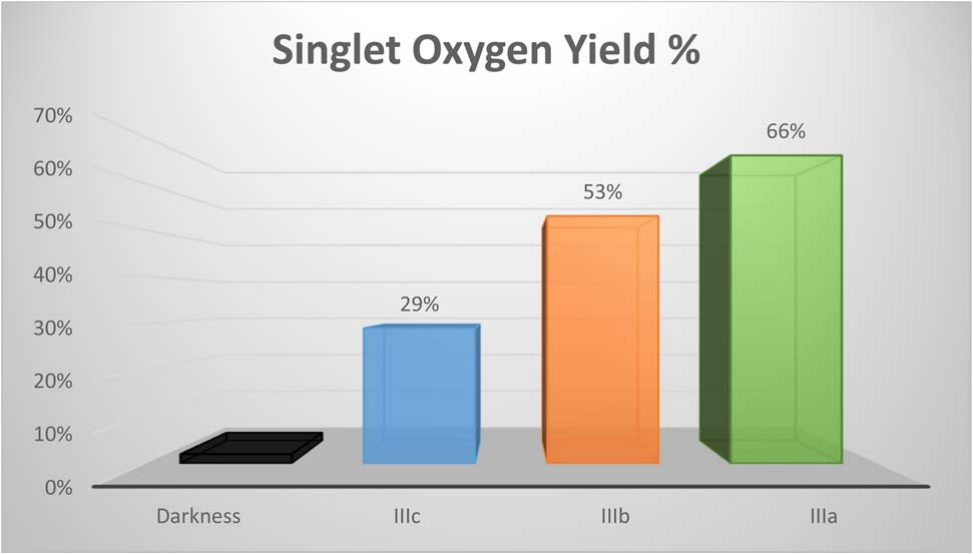
Percentage of singlet oxygen efficiency of PDIs.

In this study, we present the synthesis and characterization of three new perylene diimide dyes. These dyes have absorptions in the range of 650–700 nm. Thus, they are readily excitable in the therapeutic window. We demonstrated and compared the relative singlet oxygen generation efficiencies of these novel PDI dyes. To that end, we studied the degradation of 1,3-diphenyl-*iso*-benzofuran (dpbf) as a singlet oxygen trap in the presence of these dyes. We observed that PDI dyes do not induce any changes in the absorption spectra of the singlet oxygen trap when kept in the dark. However, when red light excitation is initiated, rapid degradation takes place. PDI dyes are not equally active in the degradation reaction. Compound 1, IIIa-1,7-dimorpholine-*N*,*N*′-(l-alanine-*t*-butylester)-3,4:9,10-perylene diimide, showed the greatest capacity for singlet oxygen generation (66%) compared to compound 2, IIIb-1,7-di-3-morpholine propylamine-*N*,*N′*-(l-valine-*t*-butylester)-3,4:9,10-perylene diimide (53%), and compound 3, IIIc-1,7-dimorpholine-*N*,*N*′-(*O-t*-butyl-l-serine-*t*-butylester)-3,4:9,10-perylene diimide (29%) (Fig. 4). Relative singlet oxygen *generation* efficiencies in isopropanol are 0.90, 0.72 and 0.39 for the sensitizers 1, 2 and 3, respectively. Also, AQYs from the soluble PDIs are 3.59, 2.88 and 1.58, respectively.

## Discussion

The three synthesized novel water-soluble green perylene diimides are efficient singlet oxygen generators, and can therefore be used as photosensitizers in PDT research. Although water solubility is a tricky problem with PDI dyes, this has been addressed successfully for these three novel green perylene diimide derivatives.

Singlet oxygen, the reactive species proposed as the most likely active agent in photodynamic therapy, was shown to be regenerated by our group by synthesizing and stimulating a continuation of green PDI dyes.

In our experiments we clearly specified the conditions required for both light and PDI dyes. Thus, we believe we may already have three examples of novel compounds of perylene diimides for photodynamic therapy. Further derivatives and targeting studies are likely to improve the effectiveness of these therapeutic agents.

## Conclusion

Three novel perylene diimides, absorbing long wavelengths, were synthesized and characterized. Bay region substitutions of the PDI systems were recently shown to display ‘improved’ absorbance characteristics and thus, similar derivatives were targeted herein. In conclusion, we have three novel dyes with absorbance in the therapeutic region of 600–850 nm (Fig. 1) and water solubility, which is a tricky problem with PDI dyes, was addressed successfully. With carboxy groups at the imide positions, and other polar groups in the bay regions, we were able to obtain up to 5 mg mL^−1^ solubility in neutral pH aqueous solutions, a remarkable feat in itself. Singlet oxygen, the reactive species proposed as the most likely active agent in photodynamic therapy, was shown to form with the excitation of green PDI dyes for the second time. The fact that this excitation is done under red light is even better, demonstrating the practical utility of this singlet oxygen generating system. These promising results are the first important step in providing new PDT agents.

## Conflicts of interest

The authors have declared no conflict of interest.

## Supplementary Material

RA-013-D3RA02338A-s001
