# Supplementary material for: Evaluation of singlet oxygen generators of novel water-soluble perylene diimide photosensitizers
Source: RSC Adv. 2023 May 22;13(23):15416–20. doi: 10.1039/d3ra02338a (PMC10201336; doi:10.1039/d3ra02338a)
Supplement: RA-013-D3RA02338A-s001 [file RA-013-D3RA02338A-s001.pdf]

Electronic Supplementary Material (ESI) for RSC Advances.
This journal is © The Royal Society of Chemistry 2023

Supporting Information for

Evaluation of singlet oxygen generators of novel water-soluble perylene diimide photosensitizers

Furkan ÖZÇİL[1], Funda YÜKRÜK[1,*]

[1]Department of Chemistry, Faculty of Arts & Science, Balikesir University, Balikesir, 10145, Turkey

Table of Contents

1. Experimental procedures

2. Characterization of the products

3. ^{1}H &^{13}C NMR spectra, mass and uv spectrums of compounds

4. The change in the absorbance pectrum of 1,3-diphenyl-*iso*-benzofuran and compound ((IIIa, IIIb, IIIc) mixture on irradiation with light (λ>600 nm).

Experimental procedures

Characterization of the products -^{1}H &^{13}C NMR spectra and mass spectrums of compounds

Synthesis of Compound I '1,7-dibromo-3,6:9,10-perylenetetracarboxylic acid dianhydride'

Compound I molecule was synthesized by adding liquid Br_2 to 3,6:9,10-perylenetetracarboxylic acid dianhydride (parent anhydride). Purification of the compound was not attempted at this stage because of the very low solubility of compound. Therefore, there were also some unbrominated and monobrominated compounds in the mixture. Parent anhydride molecule with a 98% purity, 1.47 g ($3.74x10^{-3}$ mol) was mixed with H_2SO_4 (12.01 mL) and I_2 (0.032 g). After mixing, they were stirred up until 12 h at 25 °C. The mixture of reaction was heated to 85 °C until 30 min. Liquid Br_2 6.56 g (2.1 mL) was poured to the pressure tube for 8 h after cooling to 25°C. Then the mixture was heated to 85 °C. Then 9.37 g (3 mL) Br_2 was added during 12 h. Then 1.67 mL of water was added to H_2SO_4 to decrease 86% of the concentration of H_2SO_4 during 1 h. The material obtained was cooled to 25 °C. On cooling, the precipitate was washed with g-4 glass frit. Then it was washed with (86% w/w) 15 g H_2SO_4. After washing, the precipitate was placed into 25 mL H_2O and then the mixture was stirred. The stirred mixture was filtered and then washed with water. At the end, the red precipitate was obtained 1.57 g (77% yield) and dried at 120 °C by vacuo. $C_{24}H_6Br_2O_6$.

Synthesis of Compound IIa '1,7-dibromo-*N,N'*-(L-alanine *t*-butylester)-3,4:9,10-perylene diimide'

After 0.462 g ($2.548x10^{-3}$ mol) $CH_3CH(NH_2)COOC(CH_3)_3$.HCl and 0.5 g ($0.903x10^{-3}$ mol) 1,7-dibromo-3,4:9,10-perylene tetra-carboxylic acid dianhydride were dissolved in 3 mL triethylamine, 10 mL H_20 and 10 mL n-butanol, they were stirred until 48 h at 85 °C. The solution was distilled by a rotary evaporator. The crude product was fractionated by chromatography on a column packed with silica gel 60-200 mesh by chloroform:methanol (97:3) as an eluent and dried under vacuum. The sample was identified by NMR (26% yield).

$C_{38}H_{32}Br_2N_2O_8$,

^{1}H-NMR (400 MHz, $CDCl_3$) δ 1.49 (s, 18H), 1.67 (d, J=7,09 Hz, 6H), 5.22 (m, 2H), 8.71 (d, J=8,161 Hz, 4H), 8.92 (s, 2H), 9.5 (d, J=8,16 Hz, 4H)

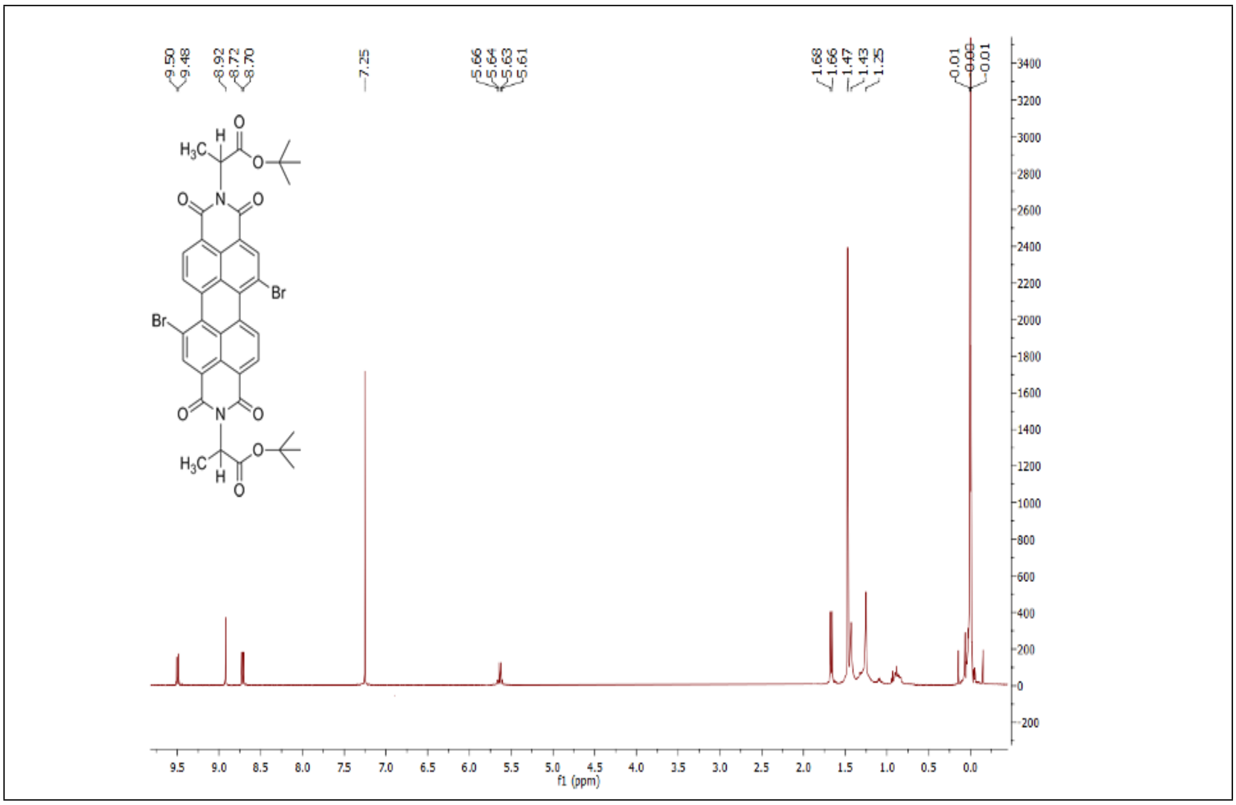

^{13}C NMR (100 MHz, $CDCl_3$) , δ[ppm], 14,79; 27,96; 50,15; 81,47; 122,72; 125,99; 129,10; 131,20; 134,17; 162,15; 168,57

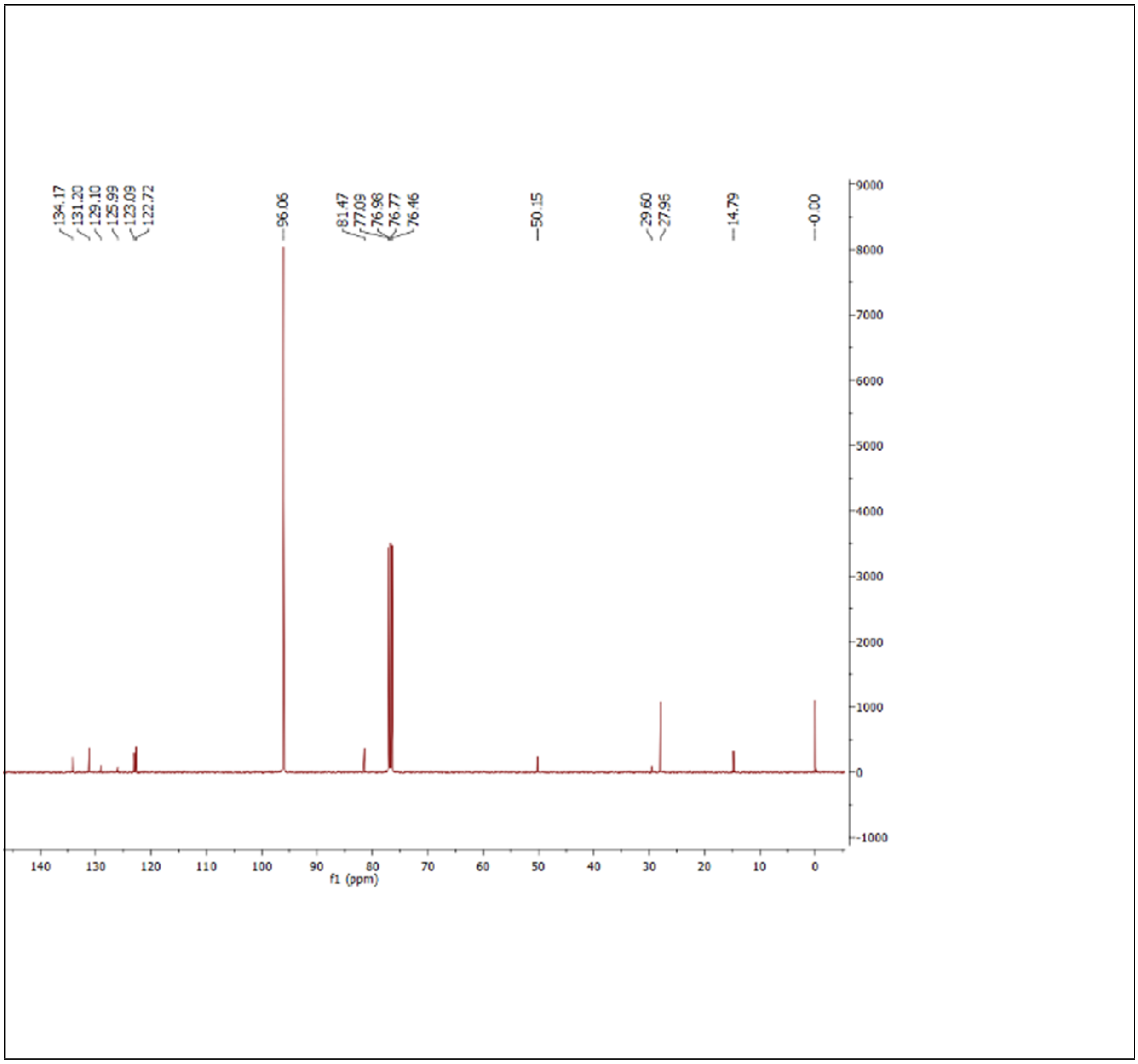

ESI-MS (m/z); 804.0496

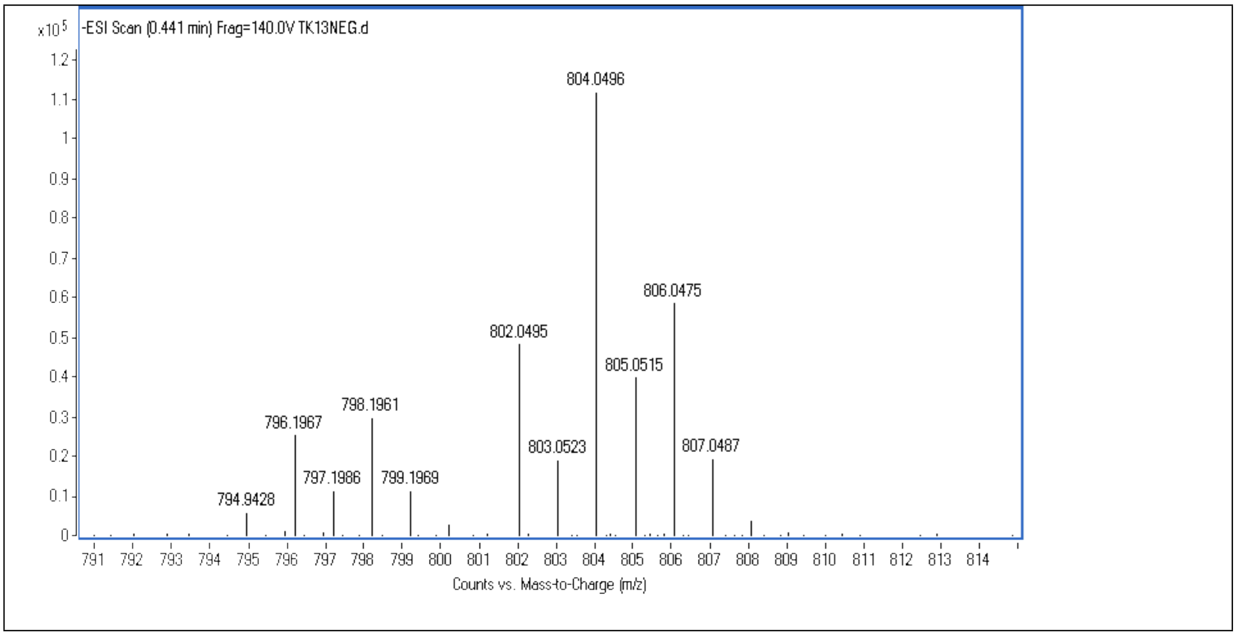

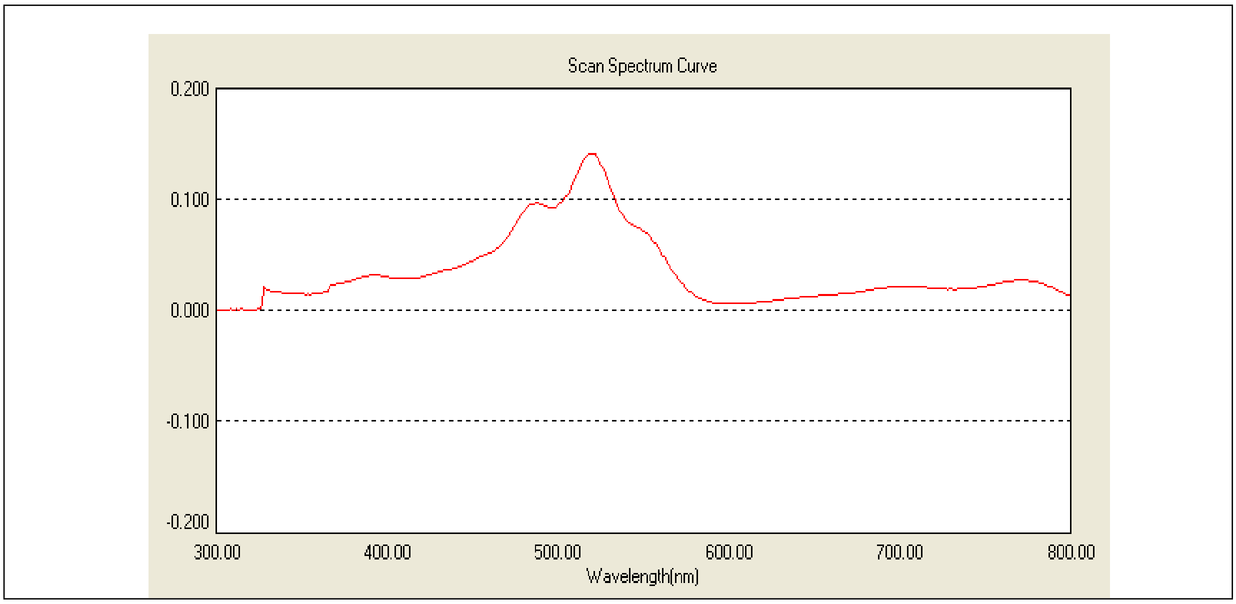

Synthesis of Compound IIIa '1,7-dimorpholine-*N,N'*-(L-alanine *t*-butylester)-3,4:9,10-perilene diimide' (3)

After 0.19 g ($2.36x10^{-4}$ mol) 1,7-dibromo-*N,N'*-(alanine *t*-butylester)-3,4:9,10-perylenediimide was stirred with 40 mL morpholine until 48 h at 85 °C, the excess morpholine was evaporated under reduced pressure. Based on the TLC analysis and column chromatography, chloroform:methanol (99:1) eluent system was chosen. The resulting reaction mixture was applied to silica gel 60-200 mesh for separation in an eluent system. The fractions on column were not separated from each other sufficiently. Accordingly, preparative TLC plates were used to separate fractions by precoated silica gel F_{254} aluminum plate (0.2 mm, Merck).

After getting the separated substance on preparative TLC plate, it was washed with chloroform:methanol (99:1) as an eluent and filtered with whatman type filter paper. The solvent of the

reaction was removed under vacuum pressure. After drying by vacuo, the sample was identified by NMR (49% yield).

$C_{46}H_{48}N_4O_{10}$

^{1}H-NMR (400 MHz, $CDCl_3$) δ 1.25 (s, 24H), 3.15 (d, J=8Hz, 2H), 3.9 (s, 8H), 5.34 (d, J=12Hz, 4H), 5.70 (d, 4H), 8.40 (s, 2H), 8.70 (s, J=8Hz, 2H), 8.35 (d, J=8Hz, 2H)

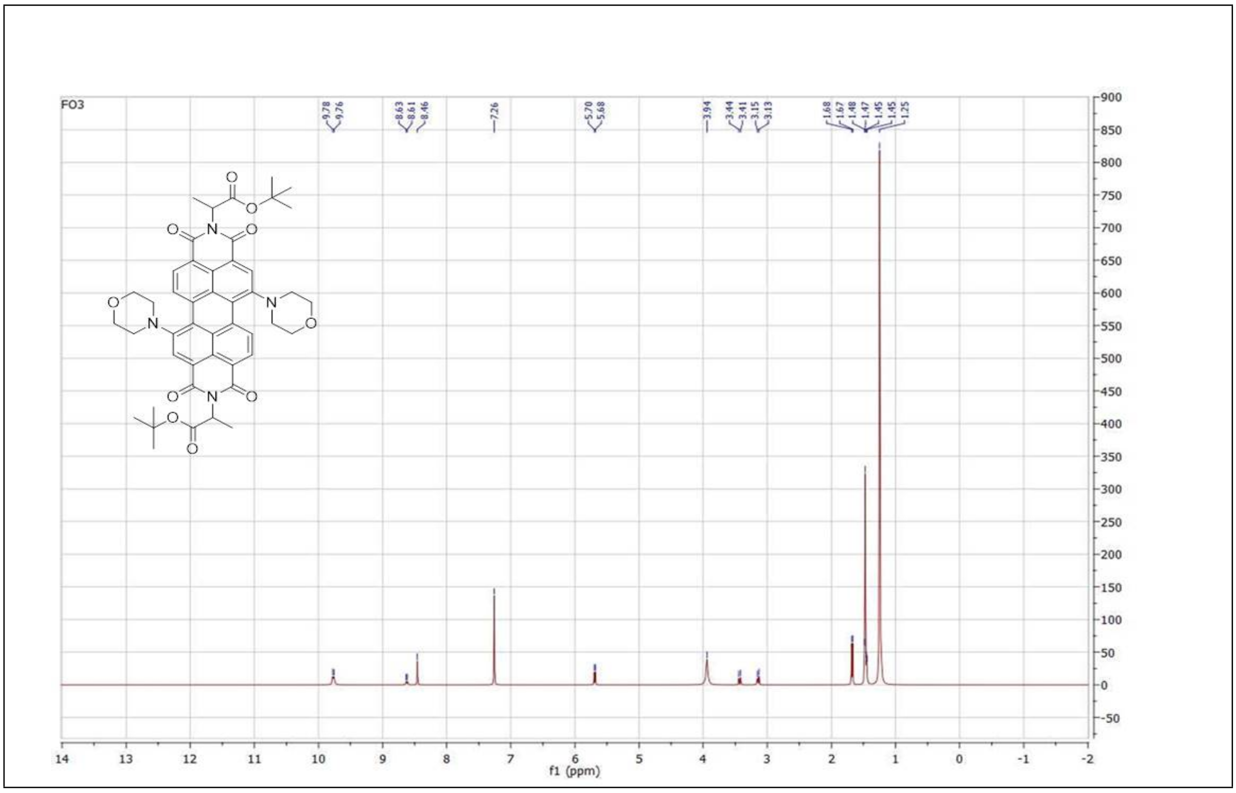

^{13}C-NMR (100 MHz, $CDCl_3$) δ 14.83, 27.97, 50.17, 51.52, 66.40, 77.00, 81.71, 122.89, 123.33, 123.84, 124.98, 128.87, 130.27, 135.46, 135.87, 150.07, 169.22

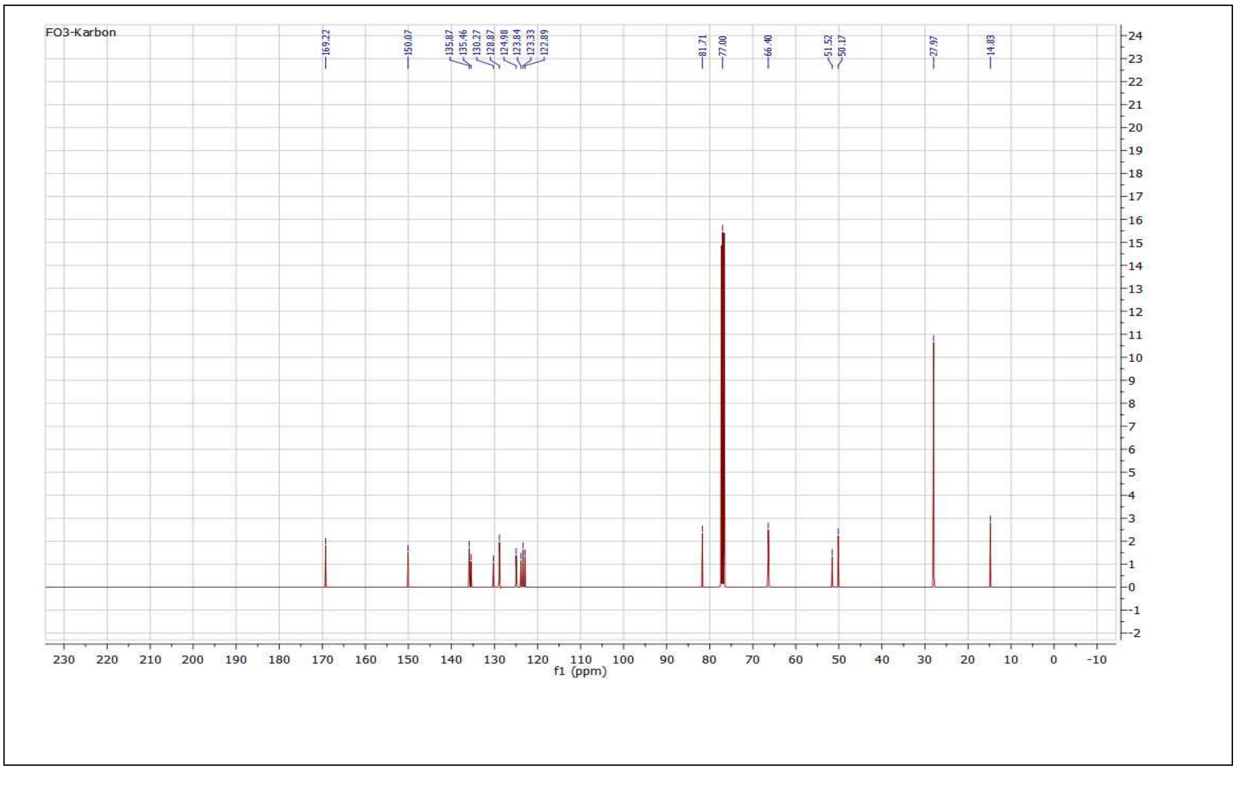

ESI-MS (m/z); 817.0

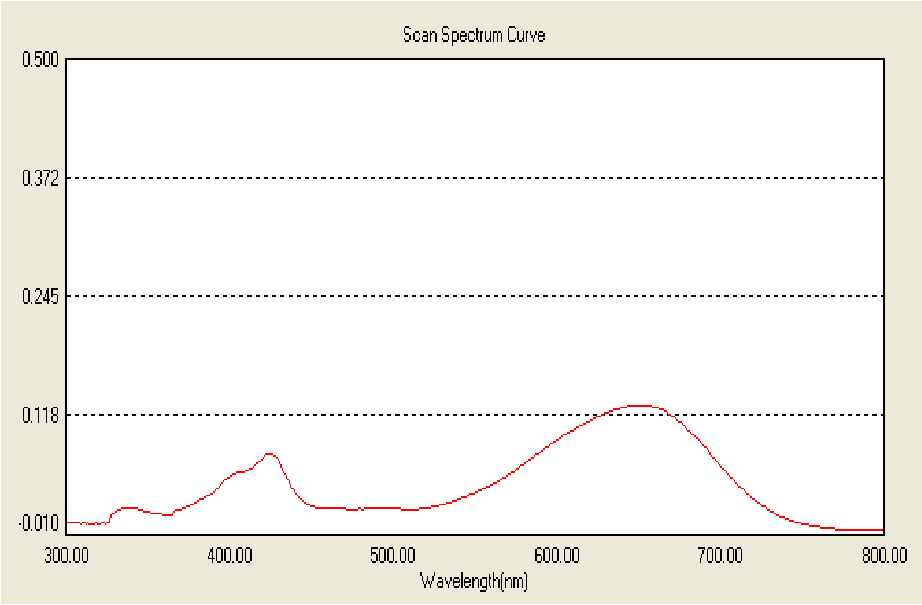

Synthesis of Compound IIb '1,7-dibromo-*N,N'*-(L-valine *t*-butylester)-3,4:9,10-perylene diimide'

After 0.534 g ($2.548x10^{-3}$ mol) $(CH_3)_2CHCH(NH_2)COOC(CH_3)_3$.HCl and 0.5 g ($0.903x10^{-3}$ mol) 1,7-dibromo-3,4:9,10-perylenetetra-carboxylic acid dianhydride were dissolved in 3 mL triethylamine, 10 mL H_20, 10 mL n-butanol and they were stirred until 48 h at 85 °C. The solution was distilled by a rotary evaporator. The crude product was fractionated by chromatography on a column packed with silica gel 60-200 mesh by chloroform:methanol (97:3) as an eluent and dried under vacuum. The sample was identified by NMR (29% yield).

$C_{42}H_{40}Br_2N_2O_8$

^{1}H-NMR (400 MHz, $CDCl_3$) δ 0.8 (s, 12H), 1.18 (s, 18H), 5.21 (m, 2H), 5.74 (m, 2H), 8.66 (d, J=8Hz, 2H), 8.88 (s, 2H), 9.45 (d, J=8Hz, 2H)

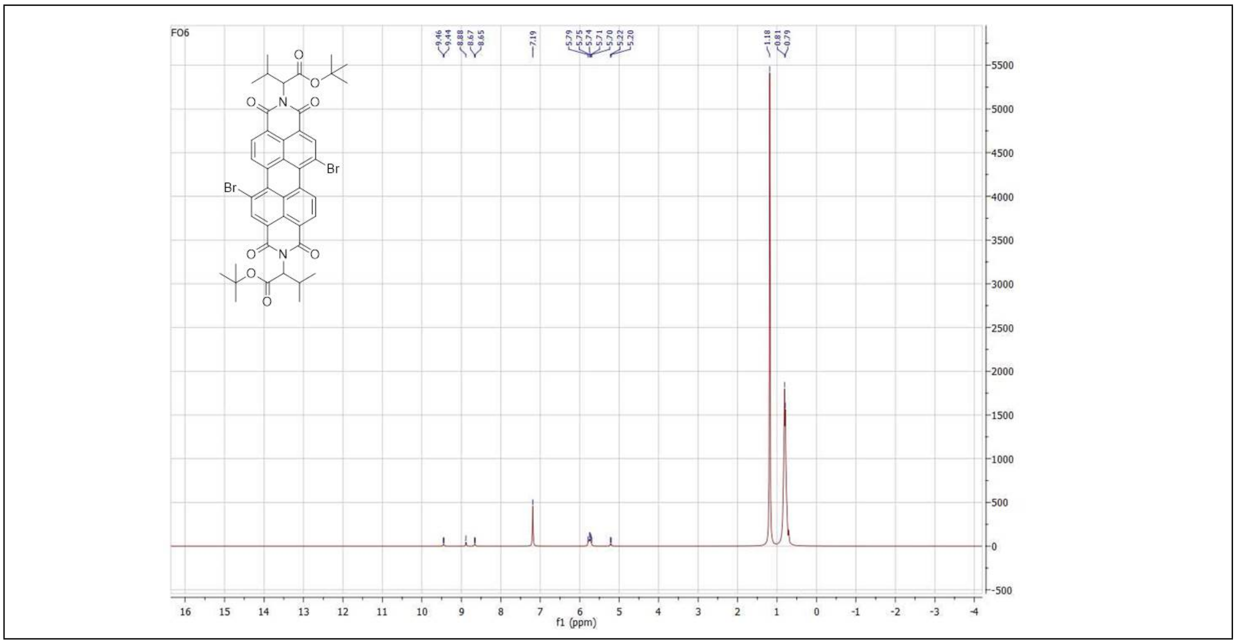

^{13}C-NMR (100 MHz, $CDCl_3$) δ 22.67, 27.97, 29.68, 59.86, 76.98, 81.79, 114.03, 115.89, 120.91, 123.49, 124.04, 130.39, 135.15, 139.25, 142.93, 162.69, 168.30

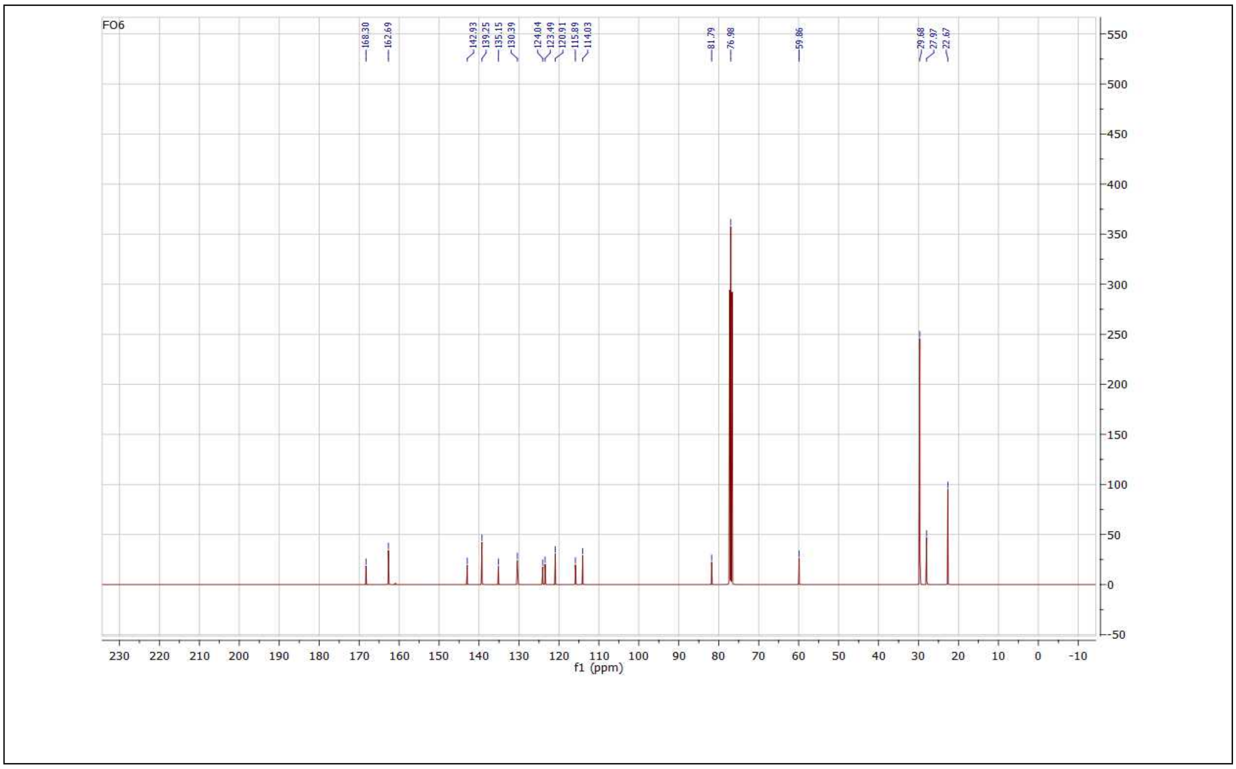

ESI-MS (m/z); 860.5

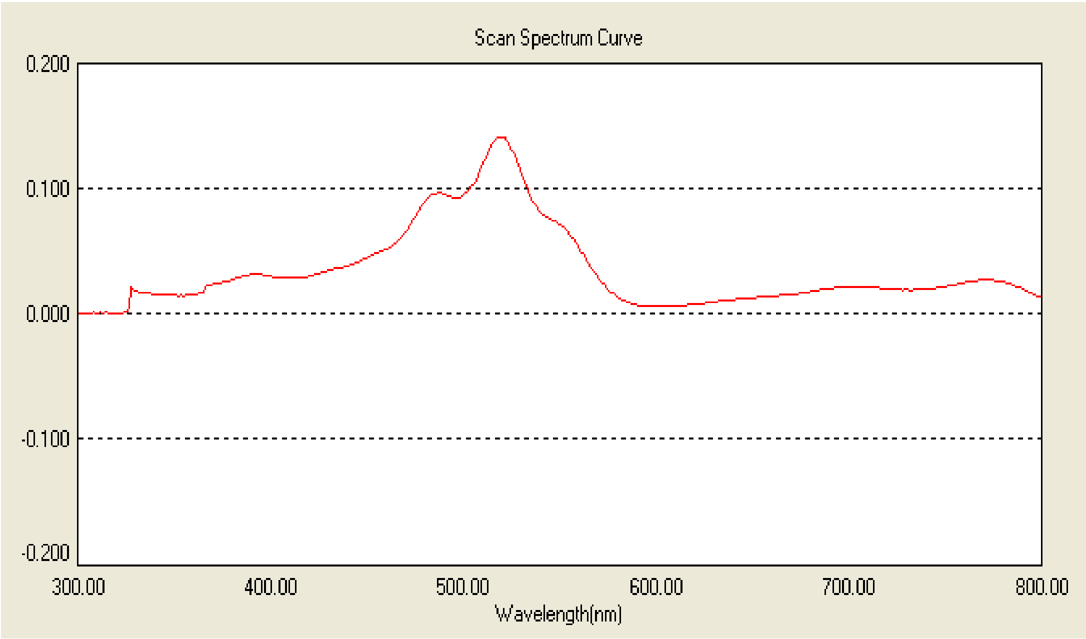

Synthesis of Compound IIIb '1,7-di-3-morpholine propylamine-*N,N'*-(L-valine-*t*-butylester)-3,4:9,10-perilene diimide' (1)

After 0.226 g ($2.626x10^{-4}$ mol) 1,7-dibromo-*N,N'*-(L-valine *t*-butylester)-3,4:9,10-perylenediimide was stirred with 40 mL N-aminopropyl morpholine in NMP until 48 h at 85 °C. The solution was evaporated under reduced pressure. Based on the TLC analysis and column chromatography, chloroform:methanol (99:1) eluent system was chosen. The resulting reaction mixture was applied to silica gel 60-200 mesh for separation in an eluent system. The fractions on column were not separated from each other sufficiently. Accordingly, preparative TLC plates were used to separate fractions by precoated silica gel F_{254} aluminum plate (0.2 mm, Merck).

After getting the separated substance on preparative TLC plate, it was washed with chloroform:methanol (99:1) as an eluent and filtered with whatman type filter paper. The solvent of the reaction was removed under vacuum pressure. After drying by vacuo, the sample was identified by NMR (49% yield).

$C_{56}H_{70}N_6O_{10}$

^{1}H-NMR (400 MHz, $CDCl_3$) δ 1.26 (s, 12H), 1.40 (s, 18H), 2.01 (m, 4H), 2,03 (m, 8H), 2.50 (m, 4H), 2.58 (m, 2H), 2.84 (m, 4H), 3.57 (s, 8H), 5.29 (d, J=8Hz, 2H), 6.47 (s, 2H), 8.24 (s, 2H), 8.35 (s, J=8Hz, 2H), 8.94 (s, J=8Hz, 2H)

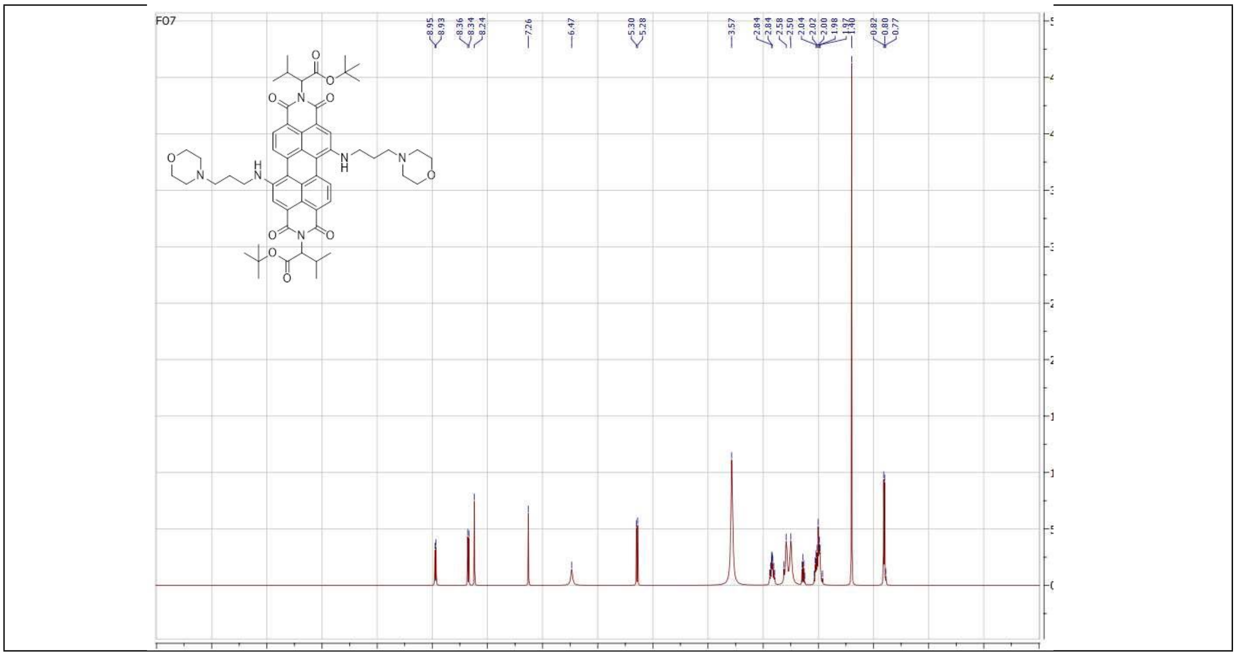

^{13}C-NMR (100 MHz, $CDCl_3$) δ 19.30, 27.96, 29.67, 43.82, 53.63, 57.18, 59.56, 66.30, 77.01, 81.38, 118.76, 119.93, 121.88, 122.43, 122.78, 127.44, 130.25, 134.32, 146.17, 163.30, 168.80

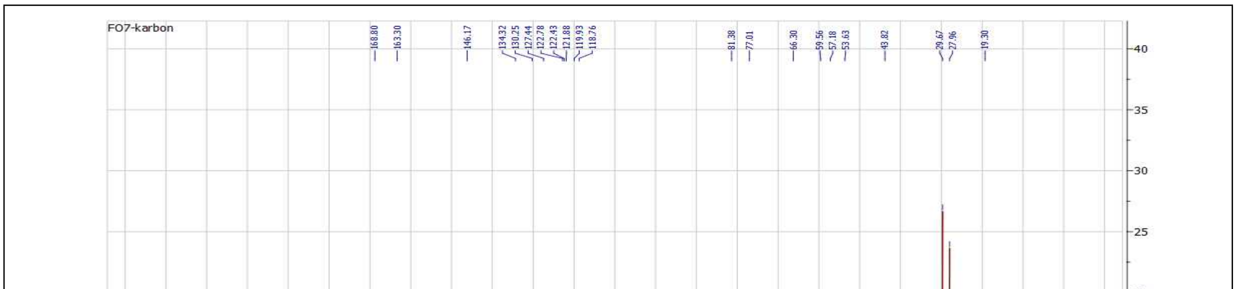

ESI-MS (m/z); 987.5

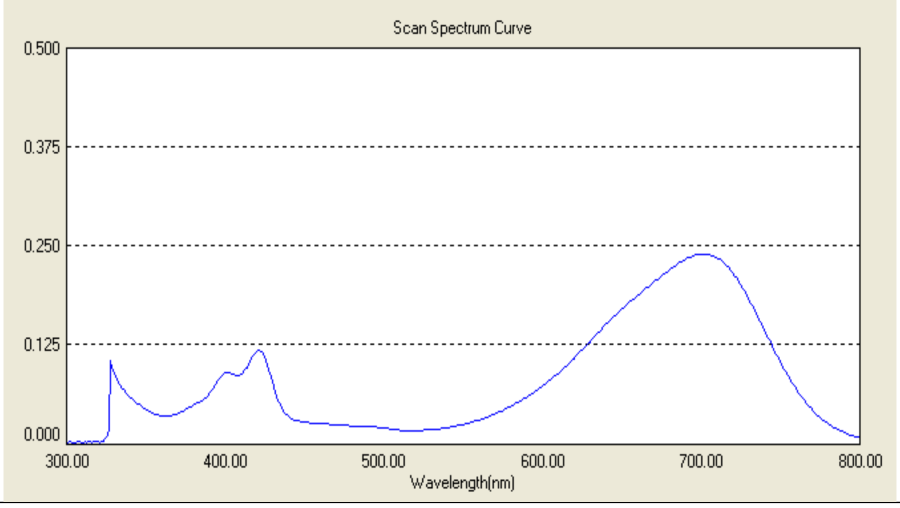

Synthesis of Compound IIc '1,7-dibromo-*N,N'*-(O-*t*-butylester-L-serine-*t*-butylester)-3,4:9,10-perilene diimide'

After 0.458 g (1.805x10^{-3} mol) $C_{11}H_{23}NO_3.HCl$ 'O-*t*-butyl-L-serine-*t*-butylester hydrochloride' and 0.5 g (0.903x10^{-3} mol) 1,7-dibromo-3,4:9,10-perylenetetra-carboxylic acid dianhydride were dissolved in 3 mL triethylamine, 10 mL H_20 and 10 mL n-butanol, they were stirred until 48 h at 85 °C. The solution was distilled by a rotary evaporator. The crude product was fractionated by chromatography on a

column packed with silica gel 60-200 mesh by chloroform:methanol (97:3) as an eluent and dried under vacuum. The sample was identified by NMR (16% yield).

$C_{46}H_{48}Br_2N_2O_{10}$

^{1}H-NMR (400 MHz, $CDCl_3$) δ 1.19 (s, 18H), 1.49 (s , 18H), 4.15 (m, 4H), 5.79 (t, 2H), 8.86 (d, J=8Hz, 2H), 8.88 (s, 2H), 9.45 (d, J=8Hz, 2H)

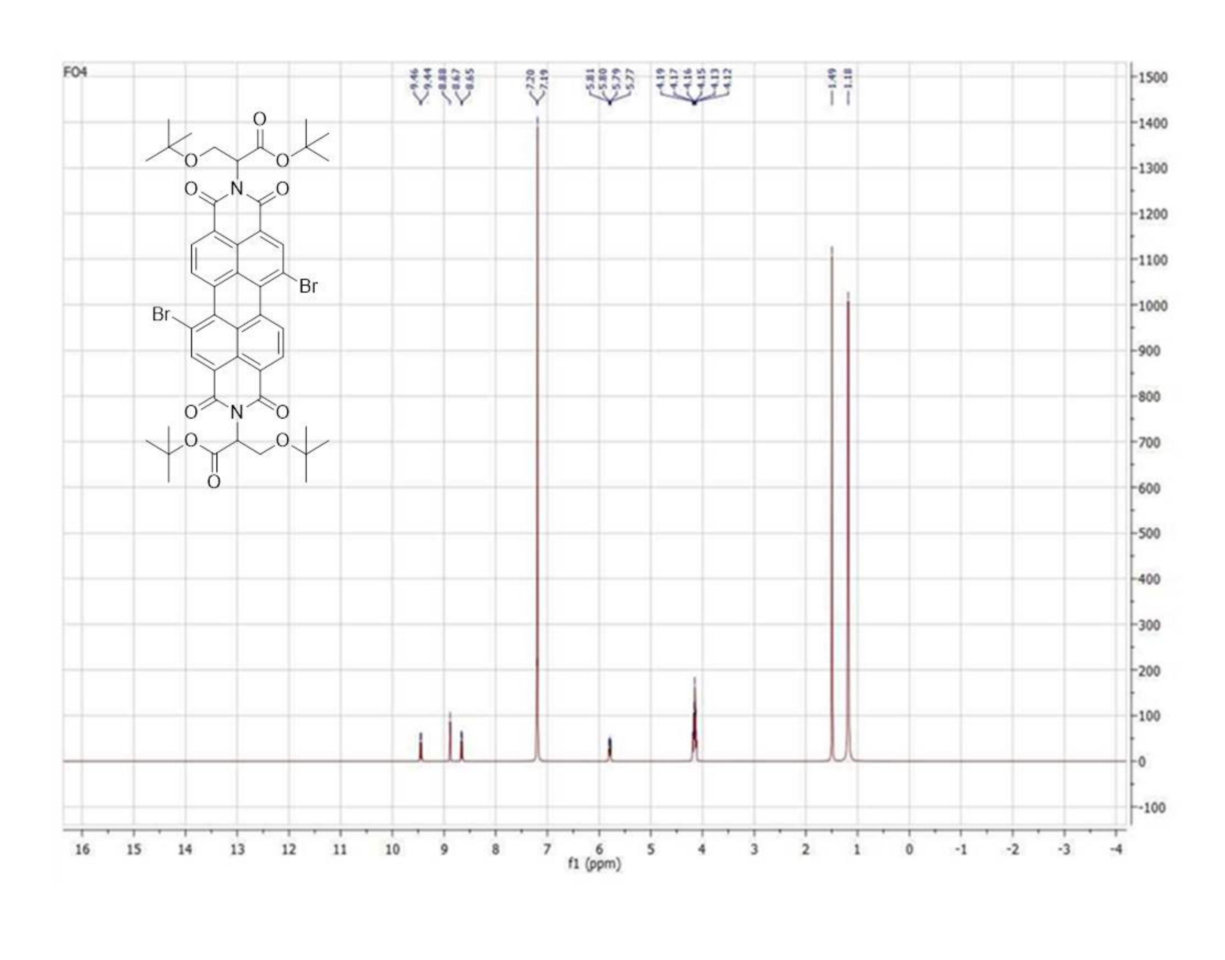

^{13}C-NMR (100 MHz, $CDCl_3$) δ 27.43, 27.97, 38.72, 68.14, 76.98, 82.35, 120.83, 121.91, 128.57, 128.77, 130.24, 130.84, 132.43, 138.18, 167.18, 167.73

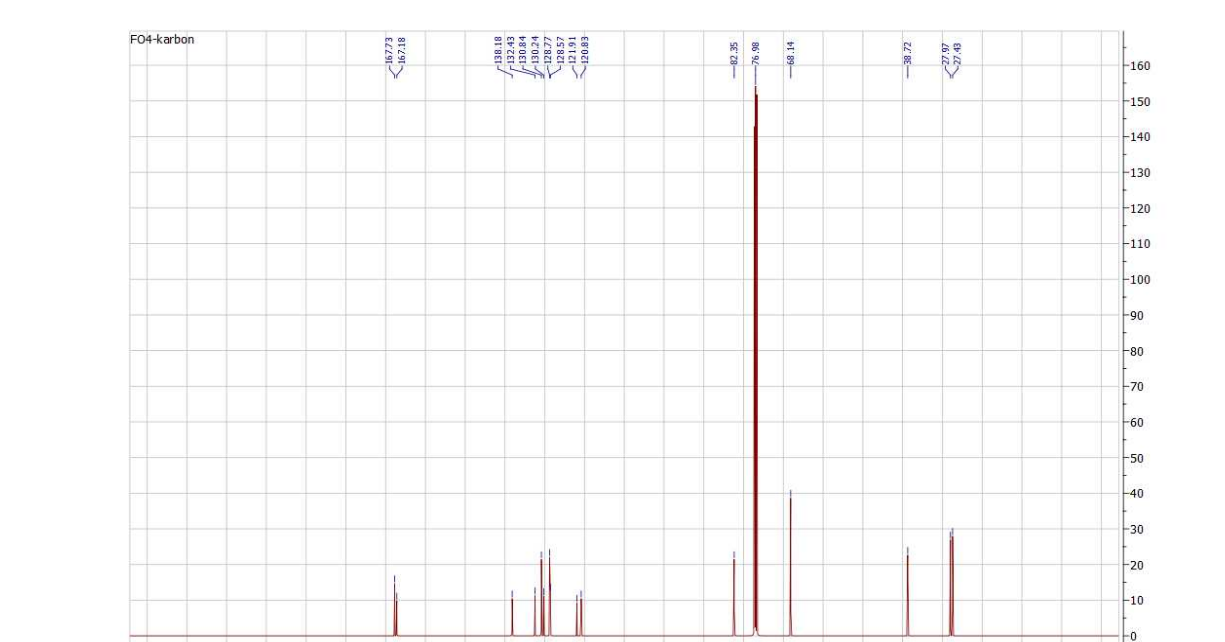

ESI-MS (m/z); 948.6

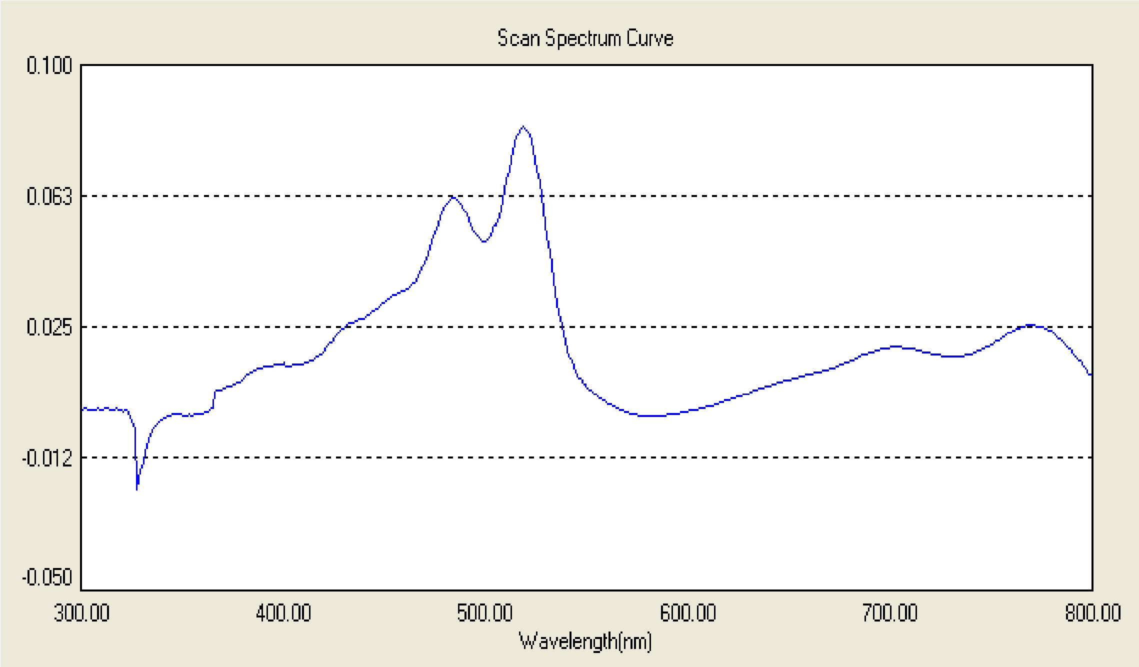

Synthesis of Compound IIIc '1,7-dimorpholine-*N,N'*-(O-*t*-butyl-L-serine-*t*-butylester)-3,4:9,10-perylene diimide' (2)

After 0.137 g (1,44x10^{-4} mol) 1,7-dibromo-*N,N'*-(O-*t*-butyl-L-serine-*t*-butylester)-3,4:9,10-perilenediimide was stirred with 40 mL morpholine until 48 hours at 85 °C, the excess morpholine was evaporated under reduced pressure. Based on the TLC analysis and column chromatography, chloroform:methanol (99:1) eluent system was chosen. The resulting reaction mixture was applied to silica gel 60-200 mesh column for separation in an eluent system. The fractions on column were not separated from each other sufficiently. Accordingly, preparative TLC plates were used to separate fractions by precoated silica gel F_{254} aluminum plate (0.2 mm, Merck).

After getting the separated substance on preparative TLC plate, it was washed with chloroform:methanol (99:1) as an eluent and filtered with whatman type filter paper. The solvent of the reaction was removed under vacuum pressure. After drying by vacuo, the sample was identifie by NMR (49% yield).

$C_{54}H_{64}N_4O_{12}$

^{1}H-NMR (400 MHz, $CDCl_3$) δ 1.09 (s, 18H), 1.47 (s, 18H), 3.15 (s, 2H), 3.46 (s, 4H), 3.93 (t, J=20Hz, 4H), 5.69 (d, J=8Hz, 2H), 8.40 (s, 2H), 8.62 (d, J=8Hz, 2H), 9.87 (d, J=8Hz, 2H)

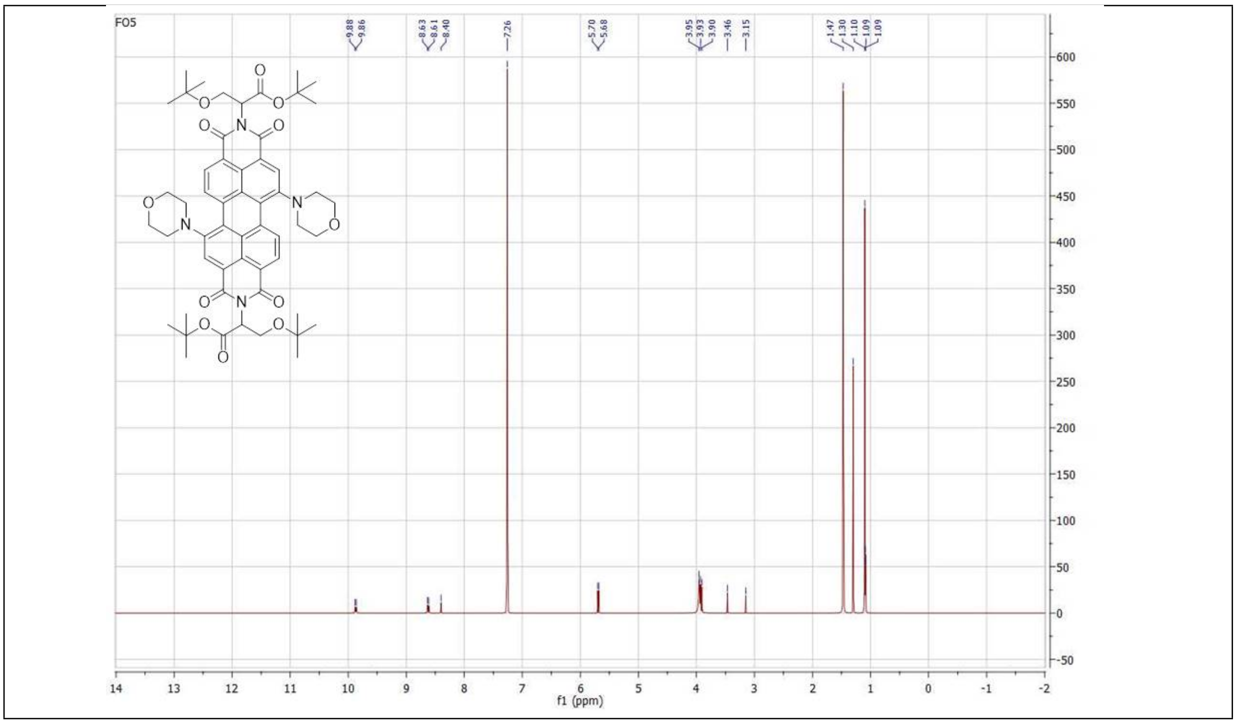

^{13}C-NMR (100 MHz, $CDCl_3$) δ 27.45, 28.00, 51.53, 62.86, 63.37, 66.45, 74.98, 82.14, 121.33, 123.37, 127.93, 128.97, 130.92, 132.37, 135.52, 150.03, 162.05, 167.54

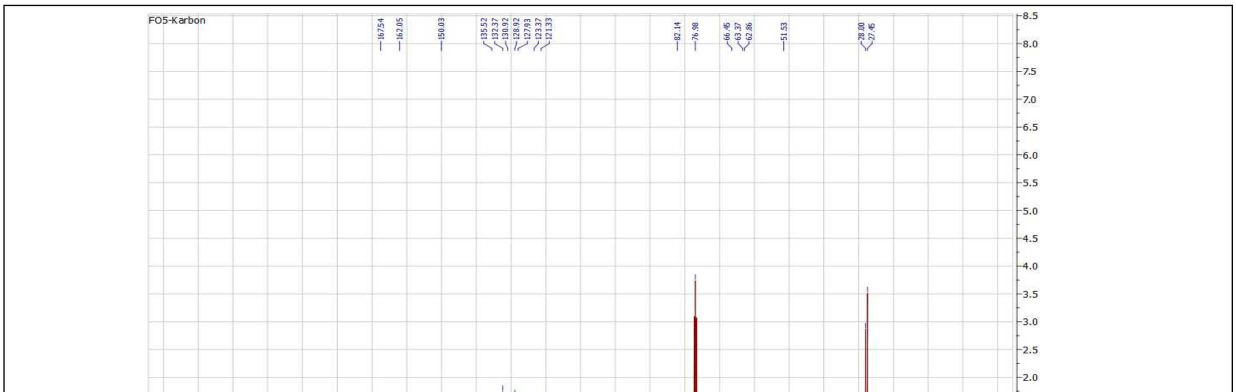

ESI-MS (m/z); 961.4

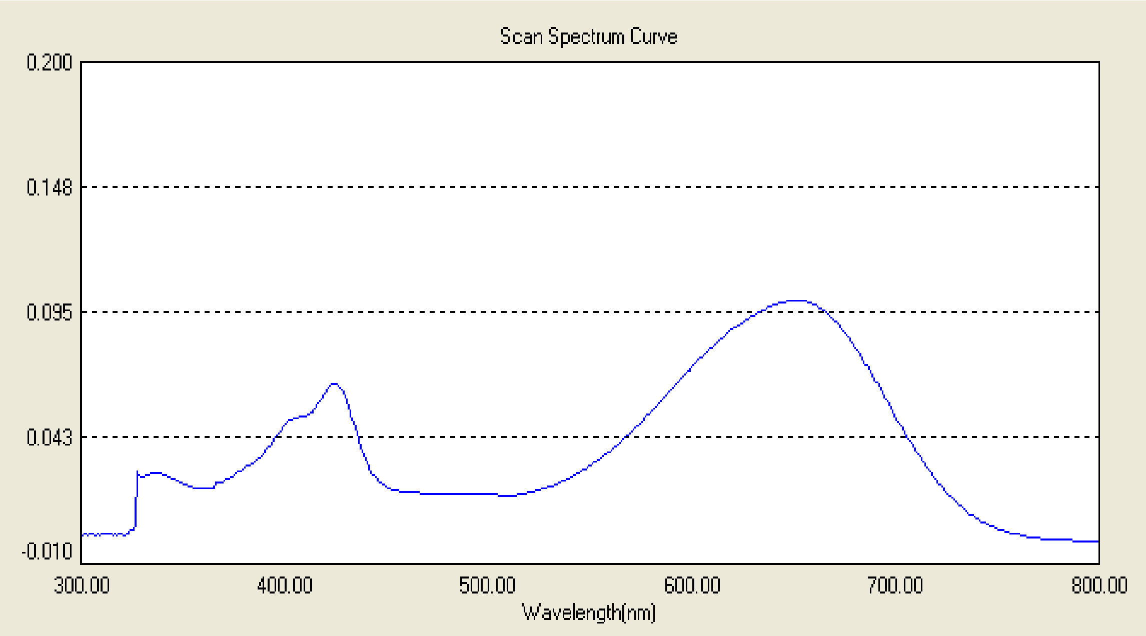

The change in the absorbance spectrum of 1,3-diphenyl-*iso*-benzofuran and compound (IIIa, IIIb, IIIc) mixture on irradiation with light (>600 nm)

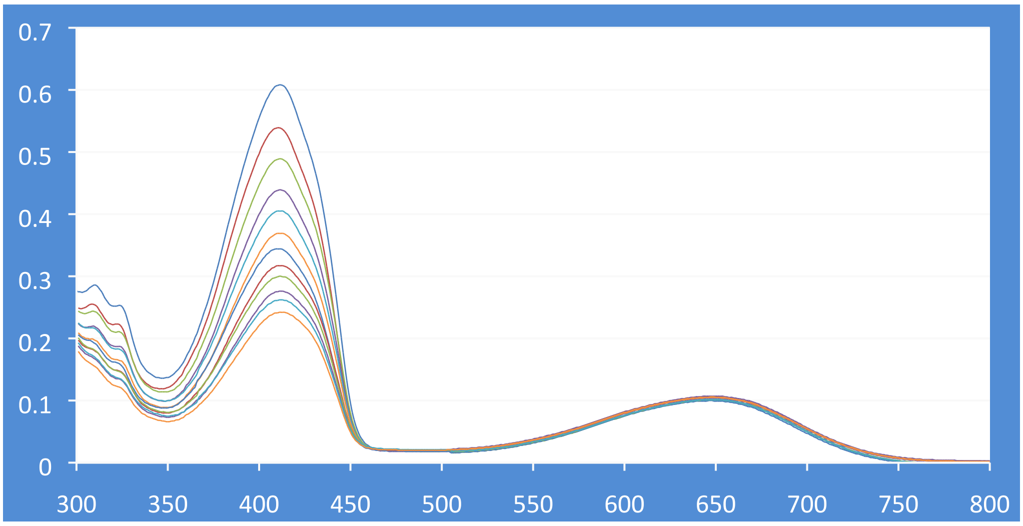
0.7
0.6
0.5
0.4
0.3
0.2
0.1
0
300
350
400
450
500
550
600
650
700
750
800

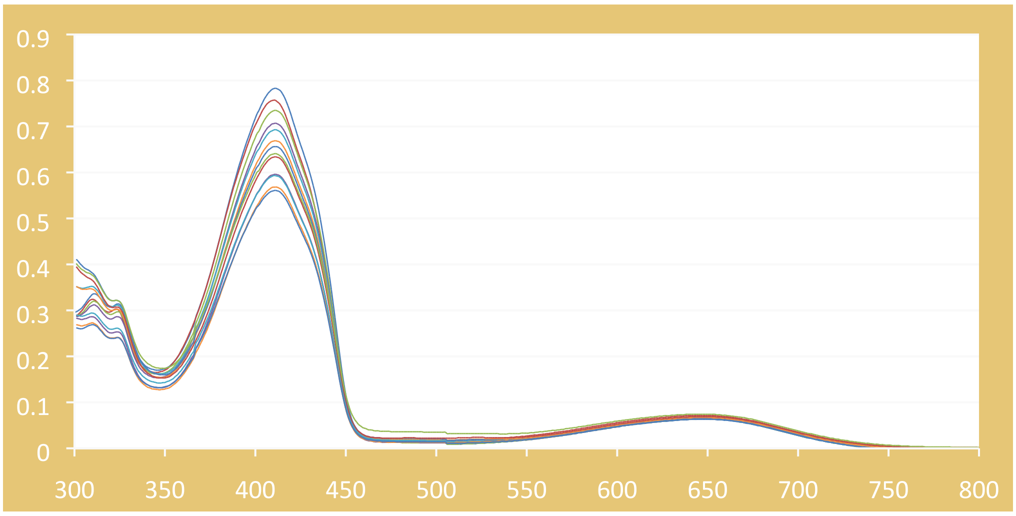
0.9
0.8
0.7
0.6
0.5
0.4
0.3
0.2
0.1
0
300
350
400
450
500
550
600
650
700
750
800

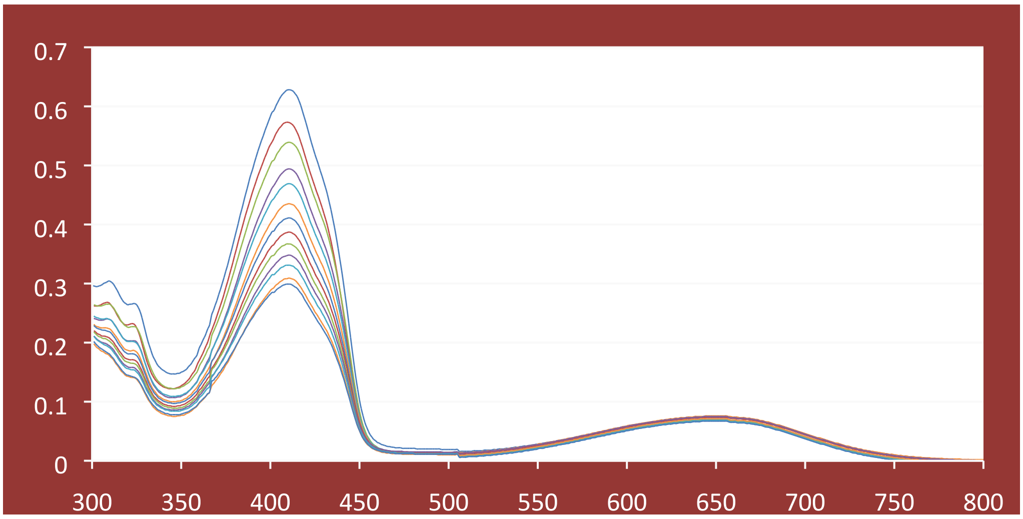
0.7
0.6
0.5
0.4
0.3
0.2
0.1
0
300
350
400
450
500
550
600
650
700
750
800

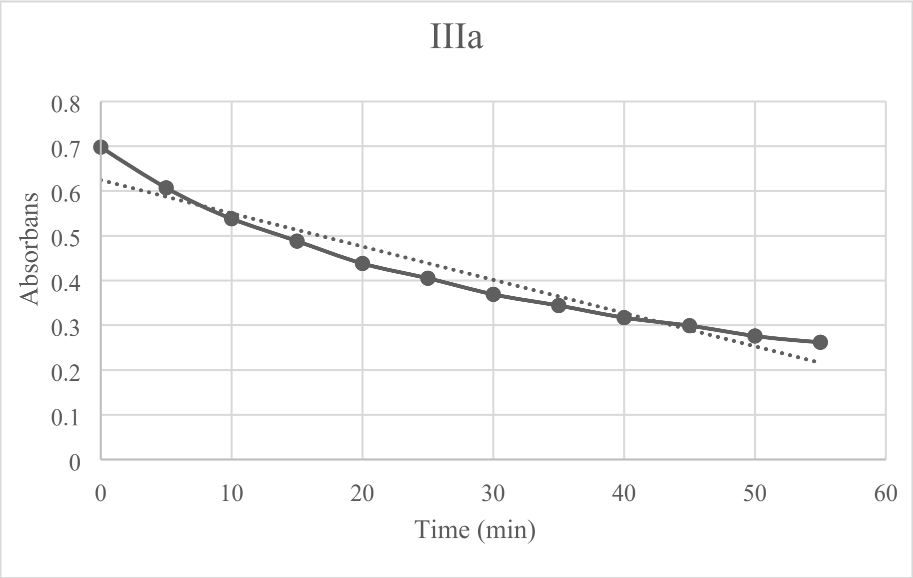
IIIa
Absorbans
0
0.1
0.2
0.3
0.4
0.5
0.6
0.7
0.8
0
10
20
30
40
50
60
Time (min)

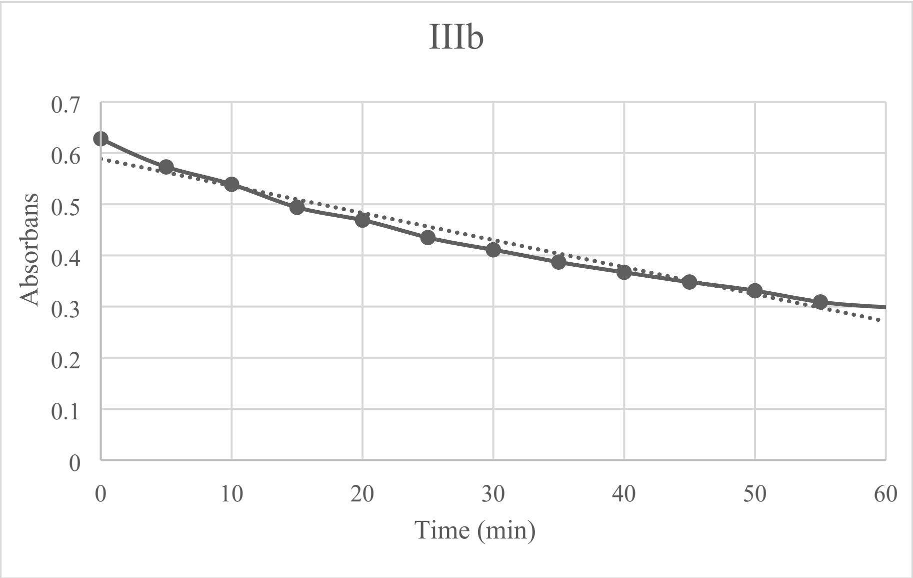
IIIb
Absorbans
0
0.1
0.2
0.3
0.4
0.5
0.6
0.7
0
10
20
30
40
50
60
Time (min)

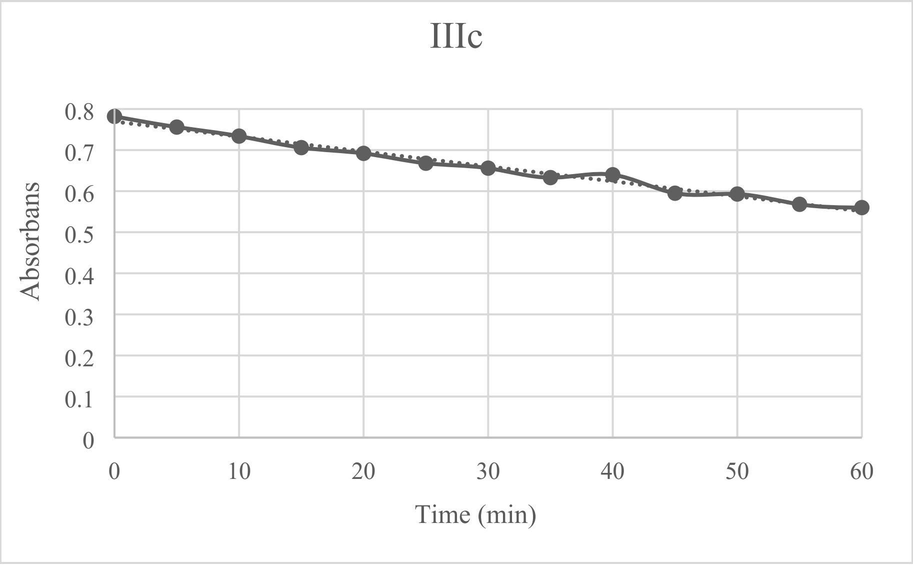
IIIc
Absorbans
0
0.1
0.2
0.3
0.4
0.5
0.6
0.7
0.8
0
10
20
30
40
50
60
Time (min)
